# Supplementary figures and images for: Non-Targeted Metabolomics of Serum Reveals Biomarkers Associated with Body Weight in Wumeng Black-Bone Chickens
Source: Animals (Basel). 2024 Sep 23;14(18):2743. doi: 10.3390/ani14182743 (PMC11429424; doi:10.3390/ani14182743)

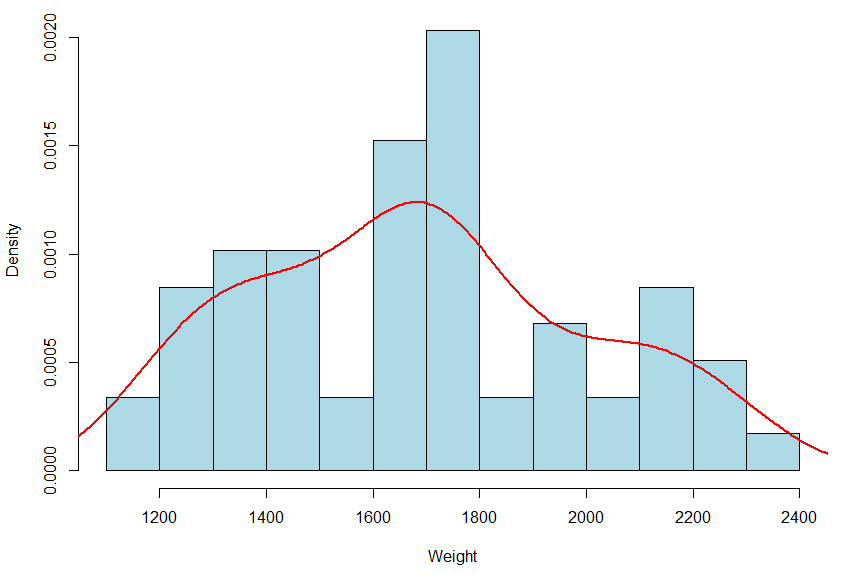

Supplement: Supplementary file 1 [file animals-14-02743-s001.zip › Supplementary Table and Figure/Supplementary Figure/Supplementary Figure1.tiff]
